# Embodied Medicine: Mens Sana in Corpore Virtuale Sano

**DOI:** 10.3389/fnhum.2017.00120

**Published:** 2017-03-16

**Authors:** Giuseppe Riva, Silvia Serino, Daniele Di Lernia, Enea Francesco Pavone, Antonios Dakanalis

**Affiliations:** ^1^Department of Psychology, Università Cattolica del Sacro CuoreMilan, Italy; ^2^Applied Technology for Neuro-Psychology Laboratory, Istituto Auxologico ItalianoMilan, Italy; ^3^Fondazione Santa Lucia, Istituto Di Ricovero e Cura a Carattere ScientificoRome, Italy; ^4^Braintrends Ltd, Applied NeuroscienceRome, Italy; ^5^Department of Brain and Behavioral Sciences, University of PaviaPavia, Italy; ^6^Department of Medicine and Surgery, University of Milano-BicoccaMilan, Italy

**Keywords:** embodied medicine, bodily self-consciousness, body matrix, predictive coding, interoception, proprioception, virtual reality, sonoception

## Abstract

Progress in medical science and technology drastically improved physicians’ ability to interact with patient’s physical body. Nevertheless, medicine still addresses the human body from a Hippocratic point of view, considering the organism and its processes just as a matter of mechanics and fluids. However, the interaction between the cognitive neuroscience of bodily self-consciousness (BSC), fundamentally rooted in the integration of multisensory bodily inputs, with virtual reality (VR), haptic technologies and robotics is giving a new meaning to the classic Juvenal’s latin dictum “*Mens sana in corpore sano*” (a healthy mind in a healthy body). This vision provides the basis for a new research field, “Embodied Medicine”: the use of advanced technologies for altering the experience of being in a body with the goal of improving health and well-being. Up to now, most of the research efforts in the field have been focused upon how external bodily information is processed and integrated. Despite the important results, we believe that existing bodily illusions still need to be improved to enhance their capability to effectively correct pathological dysfunctions. First, they do not follow the suggestions provided by the free-energy and predictive coding approaches. More, they lacked to consider a peculiar feature of the human body, the multisensory integration of internal inputs (interoceptive, proprioceptive and vestibular) that constitute our inner body dimension. So, a future challenge is the integration of simulation/stimulation technologies also able to measure and modulate this internal/inner experience of the body. Finally, we also proposed the concept of “Sonoception” as an extension of this approach. The core idea is to exploit recent technological advances in the acoustic field to use sound and vibrations to modify the internal/inner body experience.

## Introduction: Going Beyond the Physical Body and Conventional Medical Approach

According to Hippocratic physicians, the main goal of medicine was to counter diseases by aiding the natural resistance of the body to overcome the metabolic imbalance (Riva, 2016a). Since then, research in pharmacology and technology has drastically improved physicians’ ability to interact with the body. However, medicine still addresses the human body as Hippocratic physicians did thousands of years ago, i.e., as just a physical body. The interaction between the cognitive neuroscience of bodily self-consciousness (BSC) and multisensory integration (Aspell et al., 2012) with virtual reality (VR), robotics and haptics is giving a new meaning to the classic Juvenal’s latin dictum “*Mens sana in corpore sano*” (a healthy mind in a healthy body). Specifically, recent advances in VR, haptic technologies, bio/neuro-feedback and brain/body stimulation technologies provide the tools for altering the human experience of being in a body (BSC) with the goal of improving health and well-being, thereby going beyond the (mentioned) conventional medical approach of only altering our physical body (Riva, 2016a).

## The Multisensory Nature of the Body

The most basic foundations of the self are arguably housed in those brain systems that represent the body (Aspell et al., 2012). Body representation is complex and involves the encoding and integration of a wide range of multisensory (somatosensory, visual, auditory, vestibular, visceral) and motor signals (Blanke, 2012). Importantly, while external objects of perception come and go, multisensory bodily inputs are continuously present and proposed as the basis for BSC (Blanke, 2012). This multisensory representation is thought to be controlled by the “Body Matrix”—a complex network of multisensory and homeostatic brain areas whose role is to protect the body by activating perceptual and behavioral programs (effectors) when something (e.g., sensation, an injury, or a pathology) alters the body and the space around it (Moseley et al., 2012b; Gallace and Spence, 2014; Wallwork et al., 2016). According to several scholars, the body matrix sustains a multisensory representation (Blanke et al., 2015) of the space around the body (peripersonal space) that not only extends beyond the body surface to integrate both somatotopic and peripersonal sensory data (Makin et al., 2008; Serino et al., 2015) but also integrates body-centered spatial sensory data (Petkova et al., 2011; Pfeiffer et al., 2013) with an object-centered body image from vision and memory (Tsakiris, 2010; Maselli, 2015) and signals from the internal organs, such as the heart and lungs (Park et al., 2016; Tsakiris and Critchley, 2016; Tsakiris, 2017). Moreover, its contents are argued to be shaped by predictive multisensory integration (Seth et al., 2012; Suzuki et al., 2013; Talsma, 2015)—higher-order networks generate bottom-up and top-down predictions about the expected sensory inputs that are used to coordinate its contents into a coherent mental representation (Bayesian principle). Specifically, according to the recent “free-energy self” model (Apps and Tsakiris, 2014; Tsakiris, 2017), individuals process their body in a probabilistic manner as the most likely to be “me”. In this view, the experience of the body is the result of a probabilistic process associating the different unimodal properties of the body from several sensory systems: *exteroception* (the body perceived through the senses, e.g., vision and touch), *proprioception* (the sense of the position of the body/body segments originating through input of muscles and joints), *vestibular input* (the sense of motion and position of the body originating through vestibular system coding for the head position and movements) and interoception (the sense of the physiological condition of the body originating through muscular and visceral sensations or vasomotor activity).

## The Body Matrix

What is the evolutionary role of the body matrix? Apparently, the body matrix serves to maintain the integrity of the boundaries of the body at both homeostatic and psychophysiological levels (Moseley et al., 2012b). This neural network might coordinate/supervise the distribution of cognitive and physiological resources necessary to protect the body (and the space around it) and adapt it to changes in structure and orientation, as recent VR-based experimental work revealed (Llobera et al., 2013). An important effect of this control is the top-down modulation induced by multisensory conflicts (e.g., visuo-tactile) over the interoceptive homeostatic systems (Blanke et al., 2016). Besides the role of body matrix in high-end cognitive processes such as social cognition (Tajadura-Jiménez et al., 2012) it exerts a top-down modulation over basic physiological mechanisms such as thermoregulatory control (Moseley et al., 2012a). In addition to supporting this vision, a recent review by Blanke et al. (2016) underlying how experimental alterations of BSC are associated with changes at the physiological level (i.e., skin conductance response to a threat directed towards the virtual body), body temperature and pain thresholds, also indicates that “changes in BSC induced by multisensory conflicts (e.g., visuo-tactile) interact with the interoceptive homeostatic systems” (p. 330). A recent study by Finotti and Costantini (2016) further expands this vision, highlighting the existence of biochemical mechanisms accounting for the dependency of multisensory body integration and BSC on the immune system, which may have important “implications for a range of neurological, psychiatric and immunological conditions where alterations of multisensory integration, body representation and dysfunction of the immune system co-exist” (p. 1).

Gallace and Spence (2014) explained that the body matrix control over physiological functions is achieved by the connections that exist between the posterior cingulate cortex and the insula. In fact, there are a number of inhibitory connections between the insula and autonomic brain stem structures (Fechir et al., 2010). Importantly, Guterstam et al. (2015b) recently demonstrated that the posterior cingulate cortex plays a key role in integrating the neural representations of self-location and body ownership—a fundamental component of BSC.

In this view, damage, malfunctioning or altered feedback from and toward the body matrix may be involved in the etiology of different clinical conditions (Riva, 2016a), from neurological disorders like neglect (Lenggenhager et al., 2012; Bolognini et al., 2016) and chronic pain (Tsay et al., 2015; Di Lernia et al., 2016b) to psychiatric disorders like schizophrenia (Ferri et al., 2014; Postmes et al., 2014), depression (Wheatley et al., 2007; Barrett et al., 2016), depersonalization/derealization disorder (Simeon et al., 2000; Jáuregui Renaud, 2015) and eating disorders (Riva et al., 2013; Riva, 2014, 2016b; Dakanalis et al., 2016; Serino et al., 2016a).

## The Emergence of Embodied Medicine

After some seminal attempts at using a rubber hand illusion (RHI; Botvinick and Cohen, 1998) and VR to modify the experience of the body (Riva, 1998a,b; Perpiña et al., 2003), in 2007, two European teams of cognitive neuroscientists independently reported in Science (Ehrsson, 2007; Lenggenhager et al., 2007) how VR technology could be used to alter BSC (producing an out-of-body experience). Since then, different researchers have used the class of bodily illusions—having the aforementioned RHI as the prototypical paradigm (Serino and Dakanalis, 2016) to study the mechanisms behind body experience and its link with higher cognitive processes. Although this perspective article does not focus on an in-depth discussion of body illusion studies, which have recently been reviewed and summarized elsewhere (Costantini, 2014; Dieguez and Lopez, 2016; Serino and Dakanalis, 2016), it is worth noting some of these studies whose results are relevant for the topic of this article. First, it has been demonstrated that illusory ownership over an invisible body reduces social anxiety responses (Guterstam et al., 2015a). Moreover, the ownership over a dark-skinned rubber hand reduces implicit racial bias (Maister et al., 2013) while the illusory embodiment of a virtual child’s body causes implicit attitude changes (Banakou et al., 2013). Finally, and beside the view of body illusions as potential non-invasive approaches for rehabilitation with neurological and psychiatric (Costantini, 2014), it has been shown that efficient episodic-memory encoding requires perception of the world from the perspective of one’s own body (Bergouignan et al., 2014).

The approach used in the aforementioned studies creates a multisensory conflict using the exteroceptive signals of the body (touch and vision). Specifically, the experience of “being” in a different synthetic/surrogate body is achieved through the cross-modal congruence between what people feel via the somatosensory pathways and what they see in VR (Normand et al., 2011; Preston et al., 2015). To reach this goal, the required technology includes a high-end immersive VR system, a real-time motion capture and a simple haptic system integrated in a platform also able to provide physiological and brain electrical activity recordings (Spanlang et al., 2014; Castelvecchi, 2016). Currently, this set-up is still expensive, costing up to $114,000 (Castelvecchi, 2016). Moreover, the field is dominated by academic research and development with almost no technology companies translating this research into true clinical VR applications. However, as VR technology is advancing quickly, this picture is expected to change due to more user-friendly (Oculus Rift and HTC) devices, available to consumers this year, which showcase high-quality VR experiences at reasonable price points—less than $3000 for a fully configured system (Castelvecchi, 2016).

But how can we use technology to modify the contents of the body matrix? As underlined by the free-energy principle (Friston, 2010; Friston et al., 2010; Limanowski and Blankenburg, 2013), our brain tries to minimize the amount of free-energy (or “surprise”) associated with the current experience by making predictions about the sensorial consequences produced by the experienced events in the environment. In this view, the contents of the body matrix are adjusted on the basis of the (dis)agreement between the actual sensory activity and the expected inputs generated through predictive multisensory integration (Allen et al., 2016). In principle, this can be done in two ways (Limanowski and Blankenburg, 2013; O’Reilly et al., 2013):

- by changing *what is predicted* by selecting only the sensory activity that confirms the model’s predictions (as happens in the RHI). This is achieved by reallocating resources to a previously deprioritized region of space and/or re-planning a motor response to an unexpected stimulus;
- by changing the *predictions of the model* through the dynamic optimization of its parameters. However, this happens only when the level of estimation of uncertainty (Courville et al., 2006), which reflects the agent’s knowledge of the environment and can be reduced when the agent has the opportunity to make further observations of the environment, is high.

In other words, *significant prediction errors* (high surprise), which can reduce the level of estimation uncertainty, will result in strong adjustments in the internal representation to predict future events effectively (O’Reilly et al., 2013). In line with this view, a possible way of correcting a dysfunctional representation of the body and improving the old model is the use of technologies to induce a controlled mismatch between the predicted/dysfunctional model and actual sensory input (Riva, 2008, 2011; Di Lernia et al., 2016a). Some recent studies have provided scientific support to this approach. For instance, driven by the evidence that body and pain representations in the brain are multisensory and partially overlap, a recent study using VR to induce changes in BSC with the goal of modulating pain, showed that embodiment over a virtual/surrogate body can impact physiological automatic responses to noxious stimuli (Romano et al., 2016). In a more recent study, Falconer et al. (2016) used a VR body-swapping illusion protocol with a sample of depressed patients to improve their self-compassion. After three repetitions of the body swapping experience, patients achieved a significant reduction in depression severity and self-criticism. While these studies highlight embodied virtual bodies as a promising technique for future pain treatments and depression, other research provides evidence that a body-swap illusion (i.e., an illusion of body ownership over a body different from the current one) can change body perception (Normand et al., 2011), memory (Serino et al., 2016b) and affect (Preston and Ehrsson, 2014), and motivate initiation and maintenance of healthy eating behaviors even in eating disorders (Keizer et al., 2016; Serino and Dakanalis, 2016) and non-operable extremely obese patients (i.e., with body mass index (BMI) >60 kg/m^2^; Serino et al., 2016c).

## The Open Challenge: Altering the Body Matrix

Despite the aforementioned (relevant) results, we believe that the existing bodily illusions still need to be improved to enhance their capability to alter/correct pathological dysfunctions effectively in the contents of the body matrix. For example, bodily illusions are hypothesized to influence pain through “substituting” the painful body part with a virtual one (Li et al., 2011). However, a recent systematic review assessing the effects of bodily illusions on clinical pain (Boesch et al., 2016) clearly showed that exteroceptive embodiment illusions, including full body ones, do not decrease pain. This gap will be overcome by bridging existing technological advances with the cognitive neuroscience of body experience and clinical research in neurology and psychiatry. The final goal is to achieve what we propose to call “Embodied Medicine” (Riva, 2016a), i.e., the use of advanced technologies to modify our experience of being in a body to improve health and well-being.

A first issue that is not addressed in the existing body illusion protocols is the assessment of the level of surprise induced by the virtual embodiment. As already noted, if the body illusion does not produce a *significant prediction error* (high surprise), reducing the level of estimation uncertainty, it is not able to update the predictive internal models of the body matrix (O’Reilly et al., 2013). However, while some of the available studies on bodily illusions used galvanic skin response to assess the level of arousal induced by stimuli threatening the body (for example Ehrsson et al., 2008; Senna et al., 2014), none of them explicitly assessed the level of surprise in their protocols. How can we measure it? The use of eye tracking assesses pupil dilation (increased pupil diameter), a relevant marker of uncertainty and surprise (Lavin et al., 2014).

A second relevant issue is the link between surprise and updating. Even if surprise and updating are usually strongly correlated, they are distinct processes (O’Reilly et al., 2013). As underlined by O’Reilly et al. (2013), “the relationship between surprise and updating depends, among other things, on the learning rate, the degree of expected stochasticity in the environment, and the expected frequency or rate of change in the underlying environment” (p. E3661). In this view, bodily illusions have to be developed to maximize the probability of updating the predictive model by assessing and tuning these variables. Moreover, both pupil dilation (increased pupil diameter) and the activity of the anterior cingulate cortex (ACC) can be used to assess the updating of the predictive model (Behrens et al., 2007; O’Reilly et al., 2013). Preliminary results of a local brain activity (LBA) neurofeedback training of the ACC revealed more local ACC-activity after successful training. This also suggests the possibility of integrating bodily illusions with a LBA-feedback protocol targeting this area to further improve the updating process (Radke et al., 2014).

Finally, to date, most of the research effort, also from the technological point of view, has addressed how external information from the body is processed and integrated and contributes to our sense of self. Notwithstanding the success of such advances, what makes our body so special is that, unlike other physical objects, not only do we perceive it through external senses (exteroception) but we also have an internal access to it through inner (interoceptive, proprioceptive and vestibular) signals. So, a future challenge is to bridge VR with bio/neuro-feedback and brain/body stimulation technologies also able to measure and modulate the internal/inner body experience. For example, Suzuki et al. (2013) created a “cardiac RHI” in which a computer-generated augmented-reality with feedback of interoceptive (cardiac) information facilitated the online integration of exteroceptive and interoceptive signals.

At present, different companies are also working in this direction. For instance, Doppel^1^, a UK SME, developed a wearable technology able to alter the heart rhythm by providing a customized haptic feedback to the wrist. The device is based on the concept of “entrainment”—a process by which people innately respond to external rhythms by auto-adjusting their heart rate to synchronize with the beat. Here, we propose the concept of “Sonoception” as a possible extension of this non-invasive approach. The core idea is to exploit recent technological advances in the acoustic field to use sound and vibrations to modify the internal/inner body experience.

## Sonoception: Using Sound and Vibration to Modify the Inner Body

Although academic and professional institutions have been slow to recognize the emergence of acoustics as a technological science (Doak, 1964), there have been advances and dissemination of knowledge of sound and vibration in recent years (Brouet et al., 2016; Mitrou et al., 2017). Sound and vibration are two, highly interrelated physical phenomena; sound is a form of energy generated by vibrations and, in turn, vibration is an oscillatory motion. Sound and vibration can affect the human body and its well-being through mechanoreceptors (receptors specialized in sensing mechanical forces) which translate the sensory input into specific somatosensory experiences due to their different threshold sensitivity to vibration (Guignard, 1971). For example, although it is well-known that the heart is sensitive to both external and internal mechanical forces, only recently have several scholars explored the subtle effects of force on cardiac function and its relevance for pathology by linking cardiovascular mechanotransduction to the arterial myogenic response (Sharif-Naeini et al., 2010; Zamir et al., 2012). Moreover, it is well known that both sound and vibration cause fluid pressure waves in the inner ear that can induce vertigo and vestibular disorders (Dix and Hallpike, 1952). Finally, the stimulation of different esophageal mechanoreceptors mediate different sets of reflexes through the activation of different sets of medullary vagal nuclei (Lang et al., 2011). Again, esophageal sensory nerves play a key role in esophageal functional disorders, chronic unexplained symptoms that have no detectable structural, inflammatory, or metabolic disease (Sengupta, 2006). These examples suggest a direct link between sound and vibration, somatosensory experiences and different diseases through the mediation of mechanoreceptors.

**Figure 1:**
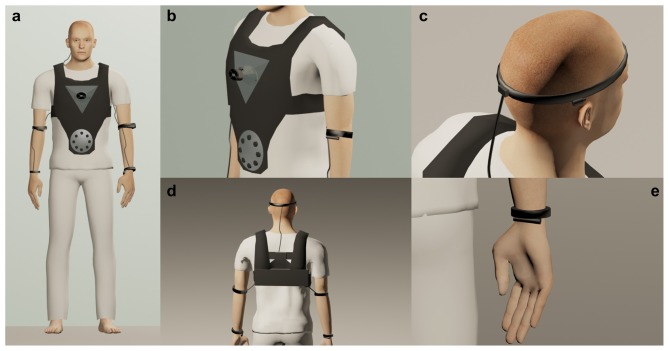
**The technology used by Sonoception. (A)** A novel non-invasive technological paradigm using wearable acoustic and vibrotactile transducers. This approach is able to modulate the inner body through the perception of movements in specific body parts. **(B)** Low Bass Frequency and Ultrasounds contactless transducers are embedded in a jacket akin to a life-vest, inducing the illusion of the perception of movements from the heart and the stomach. **(C)** A detail of a wearable linear actuator that conduces bone-vibration evoking vestibular myogenic potentials originating from selective activation of the otolithic organs. **(D)** Battery pack and electronics are hidden on the back of jacket. This system will be easy to wear and to integrate with other interfaces such as bio-signal recording and stimulation systems. **(E)** A detail of the spindle actuator applied to a wrist produces a sensation of hand displacement.

Based on this knowledge, and with the aim of s(t)imulating all the components of the inner body, the technology used by Sonoception would make use of the technology displayed in Figure 1. Specifically, (for a detailed description of the technology and rationale, see Table 1):

- For *Interoception* we will employ contactless acoustic transducers to stimulate mechanoreceptors from chest and abdomen, inducing respectively the perception of movements in the heart in the stomach. A different strategy will be employed for the two organs; while ultrasounds will be used for the stomach, we plan to use low bass frequencies for the heart.
- For *Proprioception* and the *Vestibular Input*, we will use vibrotactile transducers to stimulate mechanoreceptors placed on muscles and on otolith organs within the vestibular system.

**Table 1:** **Sonoception: rationale and technology**.

**Inner body sensory system**	**Body site**	**Technology**	**Proposed approach**
Interoception	Stomach	Ultrasound	Ultrasound waves (>20 KHz)—frequencies higher than the upper audible limit of human hearing—are often used in medicine (i.e., sonography of fetus) as totally free from side effects for human health. The ultrasonic technological devices developed for medical applications are basically used for imaging visceral anatomy. However, in recent research (Marzo et al., 2015), usage of ultrasonic transducers has been suggested as a new methodology that “can exert radiation forces and form acoustic traps at points where these forces converge permitting the levitation of particles of a wide range of materials and sizes through air, water or biological tissues” (p. 2). In this vein, holographic acoustic elements could be employed to translate the particles of food eaten with consequent motion of the stomach walls (Kang and Yeh, 2010; Hong et al., 2011).
Interoception	Heart	Low bass frequency	Bass sounds (50–120 Hz) are also prevalent in living and working environments and, despite its low audibility, low frequency noise often causes a person to experience a vibratory sensation. One of the most prominent effects of high-level low frequency sound is the so-called “chest slam”, i.e., the sensation that the chest is resonating. Studies report that pure tones with sound pressure levels of 100 dB enable the perception of chest vibration (Schust, 2004; Takahashi, 2011).
Proprioception	Muscles	Vibrotactile transducers	Cutaneous receptors in the skin around fingers, elbows, ankles and knee joints provide exteroceptive and proprioceptive information. Similar to muscle spindles, these receptors encode both movement kinematics and show directional sensitivity (Lee et al., 2013). When a vibration of approximately 70–100 Hz is applied to a tendon of the biceps or triceps muscle of a physically immobile limb obstructed from view, a sensation of arm displacement is generated (Naito et al., 1999). Notably, increasing the vibration frequency increases the velocity of the perceived illusory movement (Roll and Vedel, 1982). When the vibratory stimulation is interrupted, the spindle discharge decreases, inducing the perception that the limb is returning towards its original position.
Vestibular input	Otolith organs	Vibrotactile trasnducers	The otoliths (the utricular and saccular maculae) are the gravity sensing organs of the inner ears. Air-conducted sounds and bone-conducted vibration have been proposed as two effective methods to evoke vestibular myogenic potentials originating from selective activation of the otolithic end organs (Manzari et al., 2010). Bone-conduced vibration at frequency of 500 Hz produces consistent craniocentric whole-body responses in standing subjects (Welgampola and Day, 2006; Curthoys and Grant, 2015). The characteristics of the response are compatible with mediation by vestibular input, although the sway direction is different from that evoked by galvanic vestibular stimulation. This suggests that different patterns of input are produced by the two types of stimulation, possibly involving different proportions of afferents from the otoliths and semicircular canals. If so, bone-conducted sound, used either in isolation or combination with galvanic vestibular stimulation, may enable investigation of hitherto unexplored aspects of vestibular function in intact freely behaving human subjects.

By exploiting the technology based on the concept of Sonoception, it will be possible to modulate the inner body (including interoception, proprioception and vestibular input), to explore how these changes may affect the internal/inner subjective experience and, more importantly, to understand how variations of inner (interoceptive, proprioceptive and vestibular) signals are related to BSC. We are aware of the explorative nature of this approach but we believe that Sonoception could open novel scientific questions on the relationship between the self and inner subjective experience.

## Concluding Remarks

With these probable/proposed changes, a possible long-term goal is the reverse engineering of the psychosomatic processes. While the inter-disciplinary medical field of psychosomatic medicine explores the relationship between psychosocial and behavioral factors on bodily processes (Kiecolt-Glaser et al., 2002), embodied medicine could do the opposite, i.e., altering bodily processes to influence psychosocial and behavioral factors (Riva, 2016a).

We suggest a software module working in a closed loop (e.g., a classifier like the technologies used in the Brain-Computer Interfaces) to facilitate the integration of the external (exteroceptive) and internal/inner (interoceptive, proprioceptive and vestibular) inputs originating from the body and the environment. This software will process and classify the psychophysiological signals, which will be translated as vibratory signals and sent back to the body by the contactless acoustic transducers in real time. This approach will allow the development of a hardware/software platform bridging VR with bio/neuro-feedback and brain/body stimulation technologies and offer an integrated tool able to address all the components of our bodily experience. Nevertheless, future clinical studies are needed to identify the best protocols and combination of technological tools to transform the dictum “*Mens Sana in Corpore Virtuale Sano*” into reality. Specifically, future research should aim at exploring the psycho-physiological and neural mechanisms enabling integration between inner body signals and exteroceptive inputs in (healthy and) clinical conditions characterized by alterations of body representation and multisensory integration of bodily information, and an altered body matrix.

## Author Contributions

Professor GR conceived and developed the initial draft. SS, DDL, EFP and AD worked with Professor GR to enhance the original draft and develop it into the final draft. All authors have reviewed and approved the final manuscript as submitted.

## Funding

This article was supported by the research projects: “Unlocking the memory of the body: Virtual Reality in Anorexia Nervosa” (201597WTTM) by the Italian Ministry of Education, Universities and Research, and “High-end and Low-End Virtual Reality Systems for the Rehabilitation of Fraility in the Elderly” (PE-2013-02355948) by the Italian Ministry of Health.

## Conflict of Interest Statement

The authors declare that the research was conducted in the absence of any commercial or financial relationships that could be construed as a potential conflict of interest.

## Footnotes

^1^http://www.doppel.london/
